# Sympathetic activity and early mobilization in patients in intensive and intermediate care with severe brain injuries: a preliminary prospective randomized study

**DOI:** 10.1186/s12883-016-0684-2

**Published:** 2016-09-13

**Authors:** A. Rocca, J.-M. Pignat, L. Berney, J. Jöhr, D. Van de Ville, R. T. Daniel, M. Levivier, L. Hirt, A. R. Luft, E. Grouzmann, K. Diserens

**Affiliations:** 1 Department of Clinical Neurosciences, Neurosurgery Unit, University Hospital CHUV, Rue du Bugnon 46, 1011 Lausanne, Switzerland; 2 Department of Clinical Neurosciences, Acute Neurorehabilitation Unit, University Hospital CHUV, Lausanne, Switzerland; 3 Ecole Polytechnique de Lausanne (EPFL), Lausanne, Switzerland; 4 Department of Clinical Neurosciences, Neurology Unit, University Hospital CHUV, Lausanne, Switzerland; 5 Division of Vascular Neurology and Rehabilitation, Department of Neurology, University Hospital Zürich, Zürich, Switzerland; 6 Biomedicine Departement, University Hospital CHUV, Lausanne, Switzerland

**Keywords:** Mobilization, Catecholamines, Robotic, Neurovegetative disorders, Subarachnoid hemorrhage, Brain injuries

## Abstract

**Background:**

Patients who experience severe brain injuries are at risk of secondary brain damage, because of delayed vasospasm and edema. Traditionally, many of these patients are kept on prolonged bed rest in order to maintain adequate cerebral blood flow, especially in the case of subarachnoid hemorrhage. On the other hand, prolonged bed rest carries important morbidity. There may be a clinical benefit in early mobilization and our hypothesis is that early gradual mobilization is safe in these patients. The aim of this study was to observe and quantify the changes in sympathetic activity, mainly related to stress, and blood pressure in gradual postural changes by the verticalization robot (Erigo®) and after training by a lower body ergometer (MOTOmed-letto®), after prolonged bed rest of minimum 7 days.

**Methods:**

Thirty patients with severe neurological injuries were randomized into 3 groups with different protocols of mobilization: Standard, MOTOmed-letto® or Erigo® protocol. We measured plasma catecholamines, metanephrines and blood pressure before, during and after mobilization.

**Results:**

Blood pressure does not show any significant difference between the 3 groups. The analysis of the catecholamines suggests a significant increase in catecholamine production during Standard mobilization with physiotherapists and with MOTOmed-letto® and no changes with Erigo®.

**Conclusions:**

This preliminary prospective randomized study shows that the mobilization of patients with severe brain injuries by means of Erigo® does not increase the production of catecholamines. It means that Erigo® is a well-tolerated method of mobilization and can be considered a safe system of early mobilization of these patients. Further studies are required to validate our conclusions.

**Trial registration:**

The study was registered in the ISRCTN registry with the trial registration number ISRCTN56402432. Date of registration: 08.03.2016. Retrospectively registered.

## Background

Severe acute neurological conditions, such as subarachnoid hemorrhage, head trauma and large brain infarcts, may be associated with severe cardiovascular manifestations, such as myocardial ischemia, arrhythmias, hypertension and neurogenic pulmonary edema [1].

Patients who experience severe brain injuries are also at risk of secondary brain damage, because of delayed vasospasm and edema. Common practice in our hospital, especially in the Intensive Care Unit, is to keep these patients on prolonged bed rest in order to maintain adequate blood flow to the brain. However, the data supporting this practice are limited [2].

Autoregulation of cerebral blood flow is partly mediated by the activation of the autonomic system, mainly in the form of sympathetic effects. The autonomic system is an internal regulatory system of the central nervous network involved in visceromotor, neuroendocrine and pain mechanisms, essential for survival. The brain controls preganglionic sympathetic and parasympathetic outputs and receives visceroceptive, humoral and exteroceptive information. Both systems are activated by internal and external factors and modulate neuronal activity, cerebral blood flow and metabolism [1].

The autonomic system is critical for reflex adjustments of cardiovascular responses. It has an influence on cardiac rate and vasomotor tone, with the aim of adjusting circulatory balance [3].

The sympathetic components of the autonomic system are mainly activated in times of stress; the production of catecholamines in the adrenal glands and the sympathetic nerve endings results in a significant increase in systolic and diastolic blood pressure and heart rate [4].

It is well known that prolonged bed rest carries important morbidity, especially in the elderly, including cardiovascular, respiratory, musculoskeletal, hematological and cognitive events. The supine position decreases for instance the ventilatory volume and impairs the clearing of secretions, resulting in atelectasis and pneumonia. Immobilization results in electrolyte imbalance [2, 5].

Prolonged inactivity leads also to deconditioning. One key feature in this process could be reduced blood volume, occurring after a few days of bed rest [6]. These complications appear during the first days of bed rest and add severe problems to the already devastating neurological injury. Another aspect to consider – in the long term- is the cardiac remodeling and change in sympathetic nerve function, which can also contribute to orthostatic intolerance after long-lasting inactivity.

Many animal and human studies suggest that intermittent exposure to gravity during a long period of bed rest is sufficient to prevent deconditioning [6] and improve outcome after awakening from coma [7]. Verticalization is integrated in a neurosensorial approach in acute neurorehabilitation and improves the results of weaning training [8]. This is the reason why rehabilitation protocols should ideally begin as soon as the patient is stabilized, in order to reduce prolonged bed rest complications, stimulate the afferent sensory system and reduce spasticity in some patients.

The aim of our study was to observe and quantify the changes in sympathetic activity with gradual postural changes (Erigo®) and with leg movements alone (MOTOmed®) after prolonged bed rest. Our hypothesis is that the gradual mobilization of neurologically impaired patients with these systems avoids orthostatic hypotension with a compensatory peak of catecholamines (adrenaline, noradrenaline and dopamine) and thus is a safe method of early mobilization in patients with neurological deficits and may prevent further complications of prolonged bed rest.

The study was realized by the Acute Neurorehabilitation Unit team, in cooperation with the Intensive Care Unit, the departments of Neurology and Neurosurgery and the Biomedical laboratory of the University Hospital in Lausanne.

## Methods

### Patient population

Thirty patients were evaluated between July 2012 and September 2014 in the Intensive or Intermediate Care Unit. In our University Hospital, the decision to admit a patient in the Intensive rather than Intermediate Care Unit depends on different criteria, including the need of mechanical ventilation and of important medical support for cardiovascular and respiratory functions. In both Units, patients are monitored 24 h/24. Eighteen patients were hospitalized under Neurosurgery responsibility, 1 under Neurology responsibility, 4 in the Neurorehabilitation Unit and 7 in the Intensive Care Unit (ICU).

All patients included in this study were adults (≥18 years old), had a severe neurological injury, traumatic or non-traumatic, and had a period of bed rest of minimum 7 days before the first mobilization out of bed. Exclusion and inclusion criteria for the study are shown in Table 1.

**Table 1:** Inclusion and exclusion criteria for the study

Inclusion criteria for Groups 1, 2, 3
• Age≥18
• Severe neurological injury
• Bed rest≥7 days
• Continuous monitoring in the Intensive or Intermediate Care Unit
• Signed informed consent
Exclusion criteria for Group 2
• Amputation of a leg, with impossibility to pedal
• Trauma or previous surgery of the feet, pelvis or lumbar column
• Abdominal open wound
• Extreme obesity (BMI> 35)
• Ulcers
• Height <150cm
• Psychiatric disease or severe agitation
Exclusion criteria for Group 3
• Fixed contractions of the legs
• Weight >135 kg
• Leg length <70 cm or > 102 cm
• Bone instability
• Open ulcers or vascular disease of the legs
• Cardiac contro-indications
• Inadequate cooperation of the patient

Fourteen patients had subarachnoid hemorrhage, 4 patients had severe brain trauma, 4 patients had intra-parenchymal hemorrhage, 2 patients had an ischemic vascular accident, 3 patients had brain anoxia and 3 patients had other injuries (epilepsy in brain dysplasia; empyema; coma of undetermined origin). Demographic data of the patient population are shown in Table 2.

**Table 2:** Demographic data

Mean age in years (range)	54.2 (18–88)
Gender	Number of patients
• Male	17
• Female	13
Diagnosis	Number of patients
• Subarachnoid hemorrhage	14
• Severe brain trauma	4
• Intra-parenchymal hemorrhage	4
• Ischemic vascular accident	2
• Brain anoxia	3
• Others	3

### Mobilization protocol

This study is a single center, parallel-group study, with simple equal randomization (1:1:1). Patients were randomized into three groups of 10 patients: Group 1 “Standard Protocol”, Group 2 “MOTOmed® Protocol” and Group 3 “Erigo® Protocol”. The randomization was made by means of a computer program, which randomly coupled each patient with a number from 1 to 3, corresponding to the mobilization protocol the patient would have. These three groups are equally heterogeneous in terms of age, sex and type of pathologies of the patients included in them (Table 3).

**Table 3:** Patients’ features and their randomization

Patient	Age	Sex	Diagnositic	Mobilization protocol
1	46	M	SAH	1
2	52	M	SAH	3
3	63	M	SAH	2
4	37	M	SBT	3
5	37	M	SBT	3
6	44	M	SAH	1
7	88	F	SAH	3
8	31	F	SAH	1
9	72	F	IPH	2
10	22	M	Epilepsy	1
11	54	F	SAH	3
12	86	M	IPH	1
13	62	F	IPH	2
14	18	M	IVA	2
15	62	M	Empyema	3
16	55	M	SAH	2
17	23	F	SBT	1
18	79	F	SBT	1
19	25	F	IPH	2
20	53	F	SAH	2
21	34	M	SAH	3
22	63	M	SAH	1
23	51	M	BA	1
24	81	M	BA	2
25	85	M	Coma	3
26	42	M	IVA	2
27	75	F	SAH	3
28	64	F	BA	3
29	60	F	SAH	1
30	64	F	SAH	2

*M* male, *F* female, *SAH* subarachnoid hemorrhage, *SBP* severe brain trauma, *IPH* intra-parenchymal hemorrhage, *IVA* ischemic vascular accident, *BA* brain anoxia

MOTOmed Letto® (Reck & Co. GmbH, Germany) is an automatic system for leg mobilization in a supine position, miming a bicycle, which allows passive, active or assisted mobilization for patients on prolonged bed rest.

Erigo® (Hocoma AG, Switzerland) is a tilting table with an integrated leg movement system, which allows progressive verticalization of the patient, adjustable to patient needs and possibilities.

For all patients, we defined T0 as their time of admission to our hospital, T1 as their admission to the Intermediate Care Unit (excepted for the patients who were so far in the ICU), T2 as the moment directly before the first mobilization out of bed, T3 during the first mobilization out of bed and T4 one hour after lying in bed again. Catecholamines and metanephrines were measured at T2, T3 and T4 and cardio-respiratory parameters (blood pressure, heart rate and respiratory rate) at T1, T2, T3 and T4. For patients with SAH, a transcranial Doppler was realized at T1, T2 and T4.

All the patients were mobilized every day in bed from their admission. Patients in group 1 and 3 were mobilized in bed by physiotherapists, according to usual clinical physiotherapy standard recommendations in our hospital. Patients in group 2 were mobilized in bed by either physiotherapists and with a MOTOmed® session of almost 30 min 5 days/week. Then, after a minimum of 7 days, all the patients were mobilized out of bed.

Patients in group 1 were mobilized out of bed only by physiotherapists. The physiotherapists measured the cardio-respiratory vital signs (T2), put the patient in a sitting position on the bed for 5 min, then in an upright position (T3) and finally reinstalled him lying in bed for one hour (T4).

Patients in group 2 were mobilized out of bed by physiotherapists for the first time after the MOTOmed® session on T3.

Patients in group 3 were mobilized out of bed with a 3 step Erigo® session: during the first step (5 min) the patient was in the supine position, with the head at 0°, and the Erigo® started leg movements (T2); during the second step (30 min) the patient was progressively put in a vertical position (at 30°–50°–70° for 10 min each) while the Erigo® continued to move the patient’s legs (T2.1, T2.2 and T3); during the third step (10 min) the patient was returned to a supine position (Table 4).

**Table 4:** Measurements’ protocol and time points for Group 1, Group 2 and Group 3

	T0	T1 (Gr 1,2,3)	T2 (Gr 1,2,3)	T2.1 (Only Gr 3)	T2.2 (Only Gr 3)	T3 (Gr 1,2,3)	T4 (Gr 1,2,3)
	Admission to CHUV	Admission to Intermediate Care Unit	First mobilizationout of bed. In supine position, 15min before mobilization	After 5 min in 30° vertical position	After 5 min in 50° vertical position	After 5 min in stand position for Gr 1, 2 and in 70° vertical position for Gr 3	After 60 min in supine position
SBP	x	x	x	x	x	x	x
DBP	x	x	x	x	x	x	x
HR	x	x	x	x	x	x	x
RR	x	x	x	x	x	x	x
Catech.			x			x	x
Doppler		If SAH	If SAH				If SAH

Gr 1 (group 1): Standard Protocol; Gr 2 (group 2): MOTOmed® Protocol; Gr 3 (group 3): Erigo® Protocol

*SBP* systolic blood pressure, *DBP* diastolic blood pressure, *HR* heart rate, *RR* respiratory rate, *Catech* catecholamine

All SAH patients had multiple transcranial Dopplers, in order to monitor cerebral blood flow velocity. Data were recorded at the moment of admission to the Intensive Care Unit, just before and after mobilization. The transcranial Doppler was realized in patients in a supine position, with a Philips IU 22 echograph and a S5-2 transcranial probe.

### Catecholamine assays

Venous blood samples for catecholamines (Epinephrine (E), Norepinephrine (NE)) determination were collected in heparinized tubes (10 mL), which were immediately placed on ice. Plasma was decanted by centrifugation at 3000 rpm at 4 °C and frozen until analysis. Catecholamine concentrations were determined using tandem mass spectrometry [9].

Plasma free metanephrines (Metanephrine (M), Normetanephrine (NM)) and Methoxytyramine (MT)) were determined by tandem mass spectrometry in the same samples [10] at T2, T3 and T4 time. Reference intervals in our laboratory are: 0.02–1.23 nmol/l for Epinephrine, 0.64–6.55 nmol/l for Norepinephrine, 0.03–0.85 nmol/l for Metanephrine, 0.04–1.39 nmol/l for Normetanephrine and <0.06 nmol/l for Methoxytyramine. MT was assessed in the aim of controlling the blood sample and the laboratory analysis, as this hormone is not influenced by the mobilization procedure.

At the same time, we measured the three components of the arterial blood pressure (BP): the mean, the diastolic and the systolic arterial pressures, because they reflect differently the orthosympathetic activity.

Patients did not have coffee, tea, chocolate, citrus fruits or bananas for at least 6 h before the test, and were not taking medication interfering with catecholamine metabolism.

### Statistical analysis

Statistical procedures were performed using Matlab® R2011a (The MathWorks, Inc., Natick, Massachusetts, USA, http://www.mathworks.com) in order to test for differences for absolute and relative differences in all BP components and in hormone rates, observed before (T2), during (T3) and after mobilization (T4).

We assessed the absolute and the relative differences of the response of interest (either BP or hormonal rates for E, NE, M, NM and MT) for each mobilization procedure. Absolute difference was calculated by subtracting the values observed at two different times, and we assessed the relative difference by calculating the percentage between values also measured at two different times. Hence, for each mobilization procedure and for each response of interest, 3 groups of difference were formed (difference between T2 and T3 values, between T2 and T4 values, and between T3 and T4 values) providing together 54 data sets.

Then, we applied a normality test (Lilliefort test) on each group and the null hypothesis of normality was rejected for many data sets, therefore, a non-parametric test, the Wilcoxon signed-rank test, was preferred; the Bonferroni-Holm (BH) method was applied for the correction of multiple comparisons. Finally, statistical inference was processed by testing the null hypothesis that data in each group came from a symmetric distribution with zero median, against the alternative that the distribution did not have zero median. We also tested the null hypothesis that data in two different groups of difference were independent samples from identical distributions with equal medians, against the alternative that they did not have equal medians.

## Results

Thirty patients were enrolled and 3 had to be excluded afterwards because they received Noradrenalin treatment just before the evaluation at time point T2 (2 patients of group 1 and 1 patient of group 2).

MT concentrations were not affected by the mobilization, providing evidence that the procedure for taking blood samples and laboratory analyses remained free of technical bias. Otherwise, neither in single group testing, nor in group comparison, did T4 data provide significant differences when compared with T2 or T3 data. This may be explained by the inter-patients variability of neurotransmitter concentrations (delayed plasmatic peak, continuous production after mobilization). Therefore, we excluded all T4 data from our analysis reducing our data set to 18, which allowed us to lower the significance threshold by reducing BH correction for multiple comparisons.

**Fig. 1:**
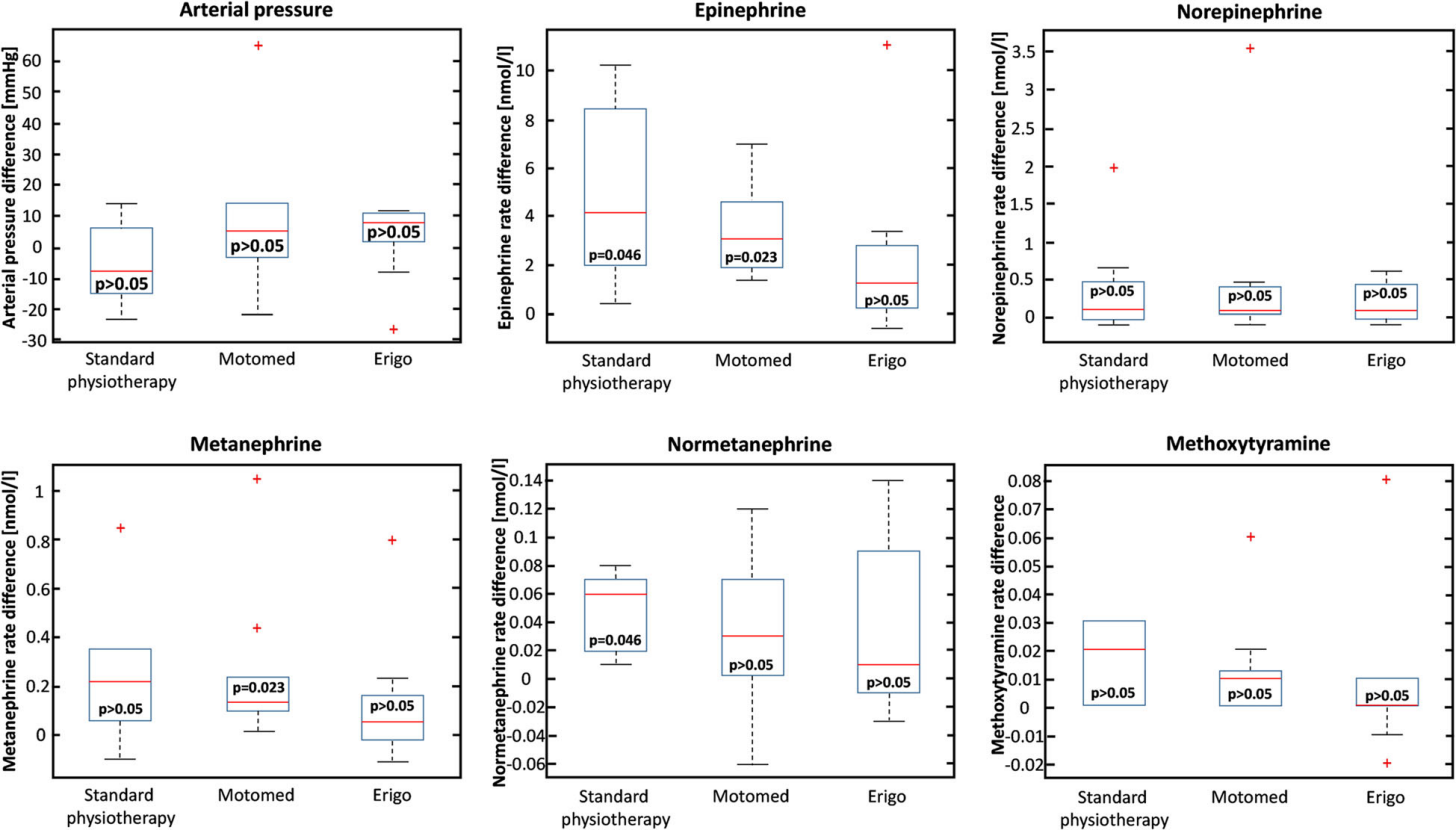
Box and whisker plots of the differences of the mean arterial blood pressure and of the neurotransmitters blood levels (epinephrine, norepinephrine, metanephrine, normetanephrine, methoxytyramine) between T2 (immediately before mobilization) and T3 (during mobilization) for the three modalities (standard physiotherapy, MOTOmed®, Erigo®). Null hypothesis of the Wilcoxon non-parametric test is tested for a symmetric distribution with zero median; significance level is 0.05 after Bonferroni-Holm correction for multiple comparisons. Legends: the red line inside the box represents the median, the blue edges of the box the 25th and 75th percentiles, the black lines the 1st and 99th percentiles, and the red crosses the outliers

In single group testing, various results were obtained with explicit homogeneity between absolute and relative differences. Firstly, no significant absolute or relative difference was observed for any of the BP components; such results may be explained by the high variability of the values measured in a small sample size. Secondly, some neurotransmitter rates changed after mobilization. Indeed, significant absolute and relative increases in E rate were measured both after MOTOmed® (identical *p* = 0.023 for slope measure and for percentage after BH correction) and standard mobilization (identical *p* = 0.046 for slope measure and for percentage after BH correction); increase of E plasma levels was higher after MOTOmed® mobilization than after standard mobilization. M plasma levels rose also significantly after MOTOmed® (identical *p* = 0.023 for absolute and relative differences after BH correction), whereas NM rates increased after standard mobilization (identical *p* = 0.046 for absolute and relative differences after BH correction). In contrast, Erigo® mobilization did not cause a significant rise of the blood level of any hormone (*p* > 0.05 for E, NE, M, NM and MT using the BH method for multiple comparison correction) in terms of absolute and relative differences. Detailed statistical results for each response of interest are presented in Fig. 1.

Group comparison did not show any significant difference irrespective of the methods. In terms of location of sample distributions for E, M and NM hormones, all value distributions are closely located together to the right of the median 0 with the Erigo® distribution situated between the median 0 and both other distributions.

## Discussion

Commonly in our hospital, severe brain injured patients are kept on prolonged bed rest because of the fear of impairing cerebral blood flow during mobilization, especially but not only in the case of SAH. On the other hand, it is well known that prolonged bed rest increases the risk of cardiovascular, respiratory and musculoskeletal problems, especially in the elderly, from as early as the first days of bed rest.

Based on this consideration, we carried out a preliminary study on severely brain-injured patients, trying to prove the safety of early mobilization in controlled conditions and its potential clinical benefits. We believe that rehabilitation protocols should ideally begin as soon as possible, in order to reduce complications, but also to stimulate the afferent sensory system in some patients. Our hypothesis was that gradual mobilization with the verticalization robot Erigo® or lower body ergometer MOTOmed® would be less stressful for these patients than simple mobilization with physiotherapists (Standard protocol) after prolonged bed rest, thus causing a lower increase in plasmatic catecholamines and less hypotensive events during mobilization.

The study was conducted on patients who had a period of bed rest of minimum 7 days before the first mobilization out of bed. The delay of 7 days was chosen for different reasons. First of all because it is the standard care for patients with severe brain injuries in our Intensive Care Unit. Then, because there are some evidences in the literature that, during the first week, the sympathetic system is overactivated in patients with cerebral tissue damages of different origin and a stable situation is restored after 7 days [11]. On the other hand, the literature shows that, after a week of bed rest, many systems start to fail (respiratory, musculoskeletal…). The idea of the study was therefore to collect data during this period of sympathetic system new stability, before the failure of other systems begins.

As reported by Yamanouchi [12] and Rössler [13], when a normal subject assumes an upright position, 500–700 ml of his blood is pooled in the lower-body vascular beds and in the splanchnic circulation. This redistribution of blood reduces central venous pressure and systemic arterial pressure, activating afferent signals of the sympathetic system. Consequently, we observe vasoconstriction, increased heart rate and an enhanced inotropic state, due to the increase in catecholamines, mainly norepinephrine [4]. All these responses are dependent on the age and physical fitness of the person, but also on their sleep-wake cycle, emotional state, attention and other factors [1].

Regarding blood pressure, some studies show that mobilization with Erigo® or MOTOmed® does not cause a significant drop in blood pressure in comparison with pre- mobilization values [14]. This could be an indirect demonstration that these systems reduce orthostatic intolerance, a well described phenomenon in the literature, due to prolonged inactivity [6].

Our statistical analysis of the blood pressure does not show any significant difference between the 3 groups. Based on our data, we cannot demonstrate any effect of the type of mobilization on the blood pressure. We can only observe a tendency towards a higher prevalence of hypotensive events in the group of patients mobilized only by physiotherapists: 6 patients of group 1 showed a hypotensive event versus 2 patients in each of the other 2 groups. The explanation for this lack of statistical significance might be due to the small sample size and the relatively high variability of the BP values in the very acute phase.

The analysis of the catecholamine results is more complex. The interpretation of a “normal sympathetic response” to stress is complicated by the fact that subtle abnormalities in autonomic control are described in many studies, as for example those occurring with smoking, [15] in diabetic patients [16] and in patients with inflammatory pathologies [17]. This could be a first problem in our study, because of the heterogeneity of our groups. In addition to this, plasma norepinephrine in venous blood depends on several factors, including local sympathetic activity and the rate of reuptake and extraction by forearm tissues. Therefore, interpretations based on forearm venous sampling of catecholamines may not necessarily reflect directly systemic autonomic activity, as reported by Vlcek et al. [4] and by Naredi et al. [18].

In 2000, Naredi et al. [18] published a very important study performed to understand the relationship between catecholamines and the risk of vasospasm in patients with SAH. They identified a dramatic elevation in sympathetic activity in patients after SAH, persisting for 7 days after the hemorrhage. The same data were observed by Takizawa et al. [19], Lambert et al. [20] and Iseda et al. [21]. Naredi also identified a relationship between sympatho-excitation and local constriction of small vessels that supply the brainstem. The possible physiopathology of this bilateral relationship is well described by Gao et al. [22]. They identified in the abrupt increase in intracranial pressure (ICP) after SAH the possible primum movens of vasospasm. The marked increase in ICP decreases cerebral perfusion pressure and results in the brainstem becoming ischemic, with lower partial oxygen pressure and the release of neurotransmitters, which induce excitation of the sympathetic center. Some other studies suggest the possibility that elevated levels of catecholamines, coupled with abnormal sensitivity of the cerebral vasculature, may be involved in the genesis of vasospasm [23, 24]. Some studies also showed that the use of norepinephrine as a hypertensive therapy post-SAH could be a potential aggravator of cerebral vasospasm [25].

Our data suggest a significant increase in epinephrine secretion during mobilization with physiotherapists alone and with MOTOmed®. This difference was confirmed by the increase of metanephrine, the O-methylated metabolite of epinephrine. No statistical changes in catecholamine production were observed during mobilization with Erigo®. There is no catecholamine that is overproduced during mobilization with Erigo® and the catecholamine levels remain stable during the rest period after mobilization.

As epinephrine is exclusively produced in the adrenal glands and reflects a response to mental or metabolic stress, its stability after Erigo® mobilization could suggest that the progressive verticalization by the robot reduces the stress emotion in these patients. The increased production of catecholamines as an indirect sign of stress was also discussed by Benarroch [1]. It means that, if we compare these three methods of mobilization, the Erigo® could be the best tolerated. There are many studies which demonstrate that patients better tolerate Erigo® than other tilt tables, in terms of greater degrees of head up tilt [26] and better stabilization of blood pressure [27] and heart rate [16]. Our hypothesis is that the key factor is the very slow and progressive mobilization allowed by Erigo®, which can be adapted to the patient response.

The most important change in catecholamine production during mobilization is observed in the group of patients mobilized with MOTOmed®. We should keep in mind this result, because of the potential risk of increasing vasospasm connected to the high levels of catecholamines [25] produced during MOTOmed® exercise in patients with SAH. On the other hand, in patients with other neurological pathologies, the stimulation of the sympathetic system should have a positive effect.

In our series, we also tried to quantify the increased risk of vasospasm during mobilization by means of trans-cranial Doppler. We only recorded one case of vasospasm, not clinically significant (in a patient mobilized by Erigo®).

Our study shows interesting results but has some limits. The first and most important one is related to the small number of included patients. This is the reason why we consider it a preliminary study. The second limit is the heterogeneity of neurological conditions we examined. Unfortunately, because of the small number of subjects, a “subgroup analysis” based on the different pathologies is impossible in our study, but could be an interesting prospective for the future. The multiplicity of factors that may influence plasma catecholamine levels could also play a role in the interpretation of our results. For example, the majority of the patients admitted to the Intensive Care Unit were under medications which could affect blood pressure and heart rate. Moreover all patients with SAH are treated in our hospital by means of hypervolemia and hypertensive therapy, in order to reduce the risk of vasospasm. All these treatments can affect sympathetic system and might have an influence on the interpretation of our data. Nevertheless, it is important to consider the absolute need of these therapies in the standard care for such patients. For this reason, getting rid of the effects caused by these treatments is extremely difficult, if Intensive Care Unit patients are included in the study. For the present study, we decided to exclude from our statistical analysis only those patients under medications which directly affect catecholamine metabolism.

## Conclusions

This preliminary prospective randomized study shows that the mobilization of patients with severe brain injuries by means of robot Erigo® does not increase the production of catecholamines. It can be considered a safe method for the early mobilization of these patients without stress, in order to reduce the risk of complications connected to prolonged bed rest.

Our study also shows that mobilization with the lower body ergometer MOTOmed® brings an important stimulation of the sympathetic system, with an increase in the production of catecholamines. For this reason, MOTOmed® should be used with caution in patients with SAH, because of the potential risk of vasospasm related to the elevation of catecholamines, but can be indicated to prevent polyneuromyopathy of critical care illness or improve the awareness of disorders of consciousness.

Because of the small number of patients included in our series, their heterogeneity and the relatively large number of outliers, further studies are required to validate our findings about the safety in the use of early robotic mobilization in the Intensive and Intermediate Care Unit.

## Abbreviations

BH: Bonferroni-Holm method
BP: Blood pressure
E: Epinephrine
ICP: Intracranial pressure
ICU: Intensive Care Unit
M: Metanephrine
MT: Methoxytyramine
NE: Norepinephrine
NM: Normetanephrine
SAH: Subarachnoid hemorrhage

## References

[1] Benarroch EE (1993). The central autonomic network: functional organization, dysfunction, and perspective. Mayo Clin Proc.

[2] Kung DK, Chalouhi N, Jabbour PM (2013). Cerebral blood flow dynamics and head-of-bed changes in the setting of subarachnoid hemorrhage. Biomed Res Int.

[3] Bahjaoui-Bouhaddi M, Cappelle S, Henriet MT (2000). Graded vascular autonomic control versus discontinuous cardiac control during gradual upright tilt. J Auton Nerv Syst.

[4] Vlcek M, Radikova Z, Penesova A (2008). Heart rate variability and catecholamines during hypoglycemia and orthostasis. Auton Neurosci.

[5] Diserens K, Moreira T, Hirt L (2011). Early mobilisation out of bed after ischemic stroke reduces severe complications but not cerebral blood flow. A randomised controlled pilot trial. Clin Rehabil.

[6] Wyller VB, Saul JP, Walløe L (2008). Sympathetic cardiovascular control during orthostatic stress and isometric exercise in adolescent chronic fatigue syndrome. Eur J Appl Physiol.

[7] Elliott L, Coleman M, Shiel A (2005). Effect of posture on levels of arousal and awareness in vegetative and minimally conscious state patients: a preliminary investigation. J Neurol Neurosurg Psychiatry.

[8] Berney L, Wasserfallen JB, Grant K (2014). Acute Neurorehabilitation: Does a neurosensory and coordinated interdisciplinary programme reduce tracheostomy weaning time and weaning failure?. NeuroRehabilitation.

[9] Dunand M, Gubian D, Stauffer M (2013). High-throughput and sensitive quantitation of plasma catecholamines by ultraperformance liquid chromatography-tandem mass spectrometry using a solid phase microwell extraction plate. Anal Chem.

[10] Grouzmann E, Matter M, Bilz S (2012). Monoamine oxidase A down-regulation contributes to high metanephrine concentration in pheochromocytoma. J Clin Endocrinol Metab.

[11] Panayiotou B, Reid J, Fotherby M (1999). Orthostatic haemodynamics responses in acute stroke. Postgrad Med J.

[12] Yamanouchi Y, Shehadeh AA, Fouad-Tarazi FM (1998). Usefulness of plasma catecholamines during head-up tilt as a measure of sympathetic activation in vasovagal patients. Pacing Clin Electrophysiol.

[13] Rössler A, László Z, Haditsch B (1999). Orthostatic stimuli rapidly change plasma adrenomedullin in humans. Hypertension.

[14] Chi L, Masani K, Miyatani M (2008). Cardiovascular response to functional electrical stimulation and dynamic tilt table therapy to improve orthostatic tolerance. J Electromyogr Kinesiol.

[15] Mancia G, Groppelli A, Di Rienzo M (1997). Smoking impairs baroreflex sensitivity in humans. Am J Physiol.

[16] Frattola A, Parati G, Gamba P (1997). Time and frequency domain estimates of baroreflex sensitivity provide early detection of autonomic dysfunction in diabetes mellitus. Diabetologia.

[17] Toussirot E, Bahjaoui-Bouhaddi M, Poncet JC (1999). Abnormal autonomic cardiovascular control in ankylosing spondylitis. Ann Rheum Dis.

[18] Naredi S, Lambert G, Edén E (2000). Increased Sympathetic nervous activity in patients with nontraumatic subarachnoid hemorrhage. Stroke.

[19] Takizawa T, Tada T, Kitazawa K (2001). Inflammatory cytokine cascade released by leukocytes in cerebrospinal fluid after subarachnoid hemorrhage. Neurol Res.

[20] Lambert G, Naredi S, Edén E (2002). Sympathetic nervous activation following subarachnoid hemorrhage: influence of intravenous clonidine. Acta Anaesthesiol Scand.

[21] Iseda K, Ono S, Onoda K (2007). Antivasospastic and anti-inflammatory effects of caspase inhibitor in experimental subarachnoid hemorrhage. J Neurosurg.

[22] Gao C, Liu X, Shi H (2009). Relationship between sympathetic nervous activity and inflammatory response after subarachnoid hemorrhage in a perforating canine model. Auton Neurosci.

[23] Athmanudh D, Botha JB, Rambiritch V (1992). Levels of catecholamines in plasma and cerebrospinal fluid in aneurismal subarachnoid hemorrhage. Neurosurgery.

[24] Lobato RD, Marin J, Salaices M (1980). Cerebrovascular reactivity to noradrenaline and serotonin following experimental subarachnoid hemorrhage. J Neurosurg.

[25] Zeiler FA, Silvaggio J, Kaufmann AM (2014). Norepinephrine as a potential aggravator of symptomatic cerebral vasospasm: two cases and argument for Milrinone therapy. Case Rep Crit Care.

[26] Luther MS, Krewer C, Müller F (2008). Comparison of orthostatic reactions of patients still unconscious within the first three months of brain injury on a tilt table with and without integrated stepping. A prospective, randomized crossover pilot trial. Clin Rehabil.

[27] Czell D, Schreier R, Rupp R (2004). Influence of passive leg movements on blood circulation on the tilt table in healthy adults [abstract]. J Neuroeng Rehabil.

